# *In vitro* evaluation of dioscin and protodioscin against ER-positive and triple-negative breast cancer

**DOI:** 10.1371/journal.pone.0272781

**Published:** 2023-02-09

**Authors:** Najat Bouchmaa, Reda Ben Mrid, Youssef Bouargalne, Sana Ajouaoi, Francesco Cacciola, Rachid El Fatimy, Mohamed Nhiri, Abdelmajid Zyad

**Affiliations:** 1 Faculty of Science and Technology, Team of Experimental Oncology and Natural Substances, Cellular and Molecular Immuno-Pharmacology, Sultan Moulay Slimane University, Beni-Mellal, Morocco; 2 Institute of Medical and Biological Sciences, Mohammed VI Polytechnic University (UM6P), Ben-Guerir, Morocco; 3 Faculty of Science and Technology, Laboratory of Biochemistry and Molecular Genetics, Abdelmalek Essaadi University, Tangier, Morocco; 4 Department of Biomedical, Dental, Morphological and Functional Imaging Sciences, University of Messina, Messina, Italy; Chung Shan Medical University, TAIWAN

## Abstract

Women’s breast cancer is one of the most significant healthcare issues for the human race that demands a proactive strategy for a cure. In this study, the cytotoxic activity (MTT assay) of two natural steroidal compounds, protodioscin and dioscin, against two major subtypes of human breast cancer estrogen receptor-positive (ER-positive)/MCF-7 and triple-negative breast cancer (TNBC)/MDA-MB-468), was assessed. The clonogenic capacity was evaluated using the clonogenic assay. Oxidative stress was determined by measuring the formation of malondialdehyde and H_2_O_2_ and the assessment of total antioxidant enzyme activities (SOD, GPx, GR, and TrxR). Protodioscin and dioscin were highly cytotoxic against the tested cell lines (1.53 μM <IC_50_< 6 μM) with low cytotoxicity on normal cells (PBMC; IC_50_ ≥ 50 μM). Interestingly, these compounds were responsible for a substantial decrease in the clonogenic capacity of both cell lines. Moreover, dioscin was able to reduce the cell motility of the invasive breast cancer cells (MDA-MB-468). At the molecular level, the two treatments resulted in an increase of reactive oxygen species. Notably, both compounds were responsible for decreasing the enzymatic activities of glutathione reductase and thioredoxin reductase. On the basis of such considerations, protodioscin and dioscin may serve as promising natural compounds to treat TNBC and ER-positive breast cancer through the induction of oxidative stress.

## Introduction

Breast Cancer is one of the most widespread cancer types in women worldwide and demands a proactive strategy for cure [1]. Breast cancer cells are subtyped as ER+/PR + HER2 - (luminal A), ER+ and/or PR + HER2+ (luminal B), ER- and PR-/HER2+ (HER2-enriched), and ER—and/or PR—HER2- (triple-negative). The luminal subtype A is the most common low-grade breast cancer subtype, with the highest survival rate, whereas triple-negative is a less common breast cancer subtype with the lowest survival rate; however, the latter is very high invasive and is difficult to cure given no hormone receptor has been discovered to target until now. Although patients with ER expressing (ER+) tumors are responsive to hormonal therapy, most ER + breast cancer patients may eventually develop resistance to hormonal therapies; on the other hand, ER- tumors are more aggressive and unresponsive to estrogen-targeted hormonal therapy [2].

Recently, research studies have been focused on the discovery of new drugs to treat breast cancer, especially triple-negative breast treatment which remains a clinical challenge [3,4]. Notably, oxidative stress can alter signaling pathways, damage DNA, and lead cellular dysfunction [5]. In cancer cells, the intracellular antioxidant capacity is mainly conferred by the glutathione and thioredoxin-dependent systems. Therefore, some studies reported that glutathione reductase (GR) and thioredoxin reductase (TrxR) enzymes might considered an effective strategy to combat tumor cells by altering their ability to eliminate ROS and cope with oxidative damage [6].

Plant-derived steroidal saponins have become a revolutionizing field as they are safe and selective as compared to conventional chemotherapies [7]. They have been widely used as therapeutic drugs against breast cancer, and they are considered suitable candidates for anticancer agent development due to their pleiotropic actions on target events in multiple manners [1].

**Fig 1:**
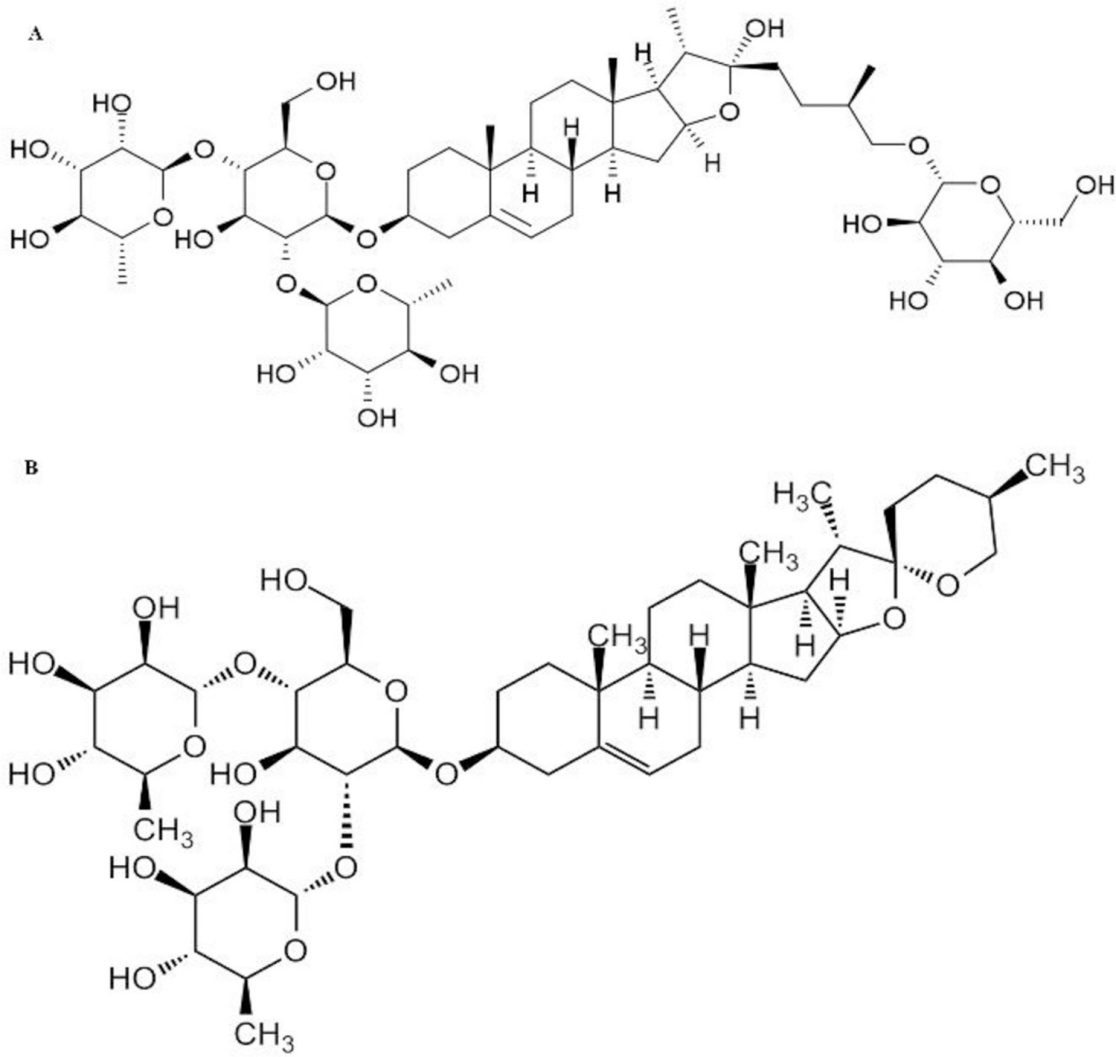
Chemical structures of protodioscin (A) and dioscin (B).

Protodioscin (Prot) and dioscin (Dios) (Fig 1A and 1B) are two typical natural steroidal saponin compounds that are derived from some medicinal plants, mainly in the Dioscoreaceae and Trigonella families [8].

These compounds have recently attracted attention because of their effectiveness in various human cancers. It has been reported that protodioscin and dioscin inhibit tumor cell survival via various mechanisms, including direct cytotoxicity [9,10], cell cycle arrest [11,12], as well as induction of apoptosis [10,13]. However, to the best of our knowledge, the anti-cancer effects of protodioscin and dioscin through ROS generation on breast cancer cells have not been illustrated so far, and the mechanisms underlying their effects still remain unknown.

In this context, the current study was designed to investigate the cytotoxicity, clonogenicity, and migration effects of protodioscin and dioscin in two molecular class subtypes, ER+ (MCF-7) and TNBC (MDA-MB-468) breast cancer cells. Furthermore, it is also discussed whether their potential involvement in molecular mechanisms related to glutathione and thioredoxin antioxidant systems.

## Material and methods

### Chemicals and reagents

RPMI 1640 medium and L-Glutamine were purchased from Lonza, Fetal Bovine Serum was from Gibco BRL (Cergy Pontoise, France), Bradford reagent was obtained from Bio-Rad (Hercules, CA, USA), and β-Nicotinamide adenine dinucleotide 2′-phosphate reduced tetrasodium salt hydrate (NADPH) from Roche Diagnostics (Mannheim, Germany). Protodioscin and Dioscin natural compounds tested in this study were purchased from Sigma Aldrich, and their purity was ≥ 99%. Cepltatin (Cisplatine) was obtained from Pharmedic Laboratories Pvt (Ltd.) Pakistan. All other chemicals and biochemical reagents were purchased from Sigma-Aldrich. Two human breast adenocarcinomas cell lines came from the Curie Institute bank (Translational Research Department, Breast Cancer Biology Team, Paris, France). They were subsequently obtained from the parent cultures of the laboratory of Experimental Oncology and Natural Substances of the Faculty of Science and Technology of Sultan Moulay Slimane University, Beni Mellal.

### Cell culture

Human breast adenocarcinoma MCF-7 (ER-positive) and MDA-MB-468 (Triple-negative human breast carcinoma) cell lines were maintained with RPMI 1640 medium supplemented with 5% heat-inactivated fetal bovine serum (FBS), 1% penicillin G-streptomycin, and 0.2% of L-Glutamine. Incubation was performed at 37°C in a humidified atmosphere containing 5% CO_2_.

### MTT assay

The human breast carcinoma cells MDA-MB-468 and MCF-7 were harvested from starting cultures at the exponential growth phase. After washing with the phosphate-buffered saline (PBS), adherent cells were harvested from sub-confluent cultures using a cell scraper and suspended in RPMI 1640 medium. The harvested cells were plated at a density of 7 x 10^4^ cells per well for MDA-MB-468 or 10^5^ cells per well for MCF-7 in flat-bottomed 96-well microplates containing 100 μL of complete medium and could adhere overnight before treatment. The cells were treated with several concentrations of protodioscin or dioscin, ranging from 0.78 μM to 50 μM, and with cisplatin (CisP) at concentrations from 0.033 to 83.02 μM. Control cells were treated with dimethylsulfoxide (DMSO) alone. The steroidal compounds were dissolved in DMSO completed with the medium. The final concentration of DMSO did not exceed 0.1%. The cells could grow for 48 h in a humidified atmosphere at 37°C and 5% CO_2_, then 100 μL of the medium was carefully removed from each well and replaced with 20 μL MTT solution (5 mg/mL PBS). As described in our previous works [5,14], after four h incubation under the same conditions, the cleavage of MTT to formazan by metabolically active cells, which were dissolved in DMSO, was quantified by scanning the plates at 570 nm using a Multiskan EX (Finland) apparatus. Three independent sets of experiments performed in duplicate were evaluated. The following equation calculated the percentage of cell viability [15]:

$$\%\;Cell\;Viability=100\;*\;A/A_{0}$$

where A_0_ and A are the absorbance of negative control and test culture, respectively.

The cytotoxic effects of protodioscin and dioscin against the breast tumor lines were compared using their IC_50_ values (concentration of tested molecules leading to 50% inhibition of cell viability).

### Cytotoxic effect against peripheral blood mononuclear cells (PBMCs)

This test was realized in order to evaluate the effect of steroidal protodioscin and dioscin against non-cancerous cells using the MTT colorimetric assay described below. To isolate the human PBMCs, blood samples were collected from healthy human donors in heparinized tubes, and the PBMCs were isolated using standard Ficoll-hypaque density centrifugation (Biomedical Research Ethics Committee Mohammed University V-Souissi Faculty of Medicine and Pharmacy of Rabat Faculty of Dental Medicine of Rabat 09/06/2014). The interface lymphocytes were washed twice with PBS. Cells were incubated in 96-well microtiter plates in the presence of the same concentrations of the steroidal compounds in the same conditions as tumor cells for 48h of incubation. As well, 20 μL MTT solution (5 mg/mL PBS) was added to the microplate; then, after four h incubation under the same conditions, the cleavage of MTT to formazan by metabolically active cells, which were dissolved in DMSO, was quantified by scanning the plates at 570 nm using a Multiskan EX (Finland) apparatus. The percentage of viability was calculated using the equation described above.

### Clonogenic formation assay

Clonogenic assay was performed on MCF-7 and MDA-MB-468 tumor cells. The cells were seeded in triplicates on 6-well plates with densities of 500 cells/well plates [16]. After overnight incubation, the cells were treated with 1 μM of protodioscin or dioscin or with 0.33 μM of the respective control CisP, then cultured in a 37°C, 5% CO_2_ incubator. The media was renewed every 3 days. After 10 days of treatment, the colonies formed were mixed with methanol/acetic acid (7:1), and stained with crystal violet (0.5% w/v) in methanol for 30 min. The survival fraction was calculated as follows: Surviving fraction = colonies counted/(cells seeded × PE) x100), where PE is the plating efficiency that represents the ratio of the number of colonies to the number of cells plated [17].

### *In vitro* wound healing test

Cell migration was assessed using an in vitro Healing test. The MDA-MB-468 cells were seeded in 6-well plates and allowed to proliferate in a confluent monolayer before serum starvation overnight. The cells were then scraped with a sterile 20 μl pipette tip and washed three times with PBS to remove floating cells. Thereafter, RPMI medium containing 2% FBS was added to each well. Cells were contacted with 1 μM of each treatment (protodioscin, dioscin, and CisP) to inhibit cell proliferation. The images were taken using an inverted microscope (Olympus) at different times: 0, 6, and 18 h. Image J analysis software was used to continuously calculate the percentage of wound closure for different types of cells. Cell migration was expressed as a percentage of wound closure relative to the initial scraped area of the wound.

### Hydrogen peroxide (H_2_O_2_) content determination

H_2_O_2_ content was determined following the protocol of Hippler et al. [18] with some modifications. Briefly, cells (0.7x10^6^ per dish) were seeded in 6-well plates and incubated in a complete medium with IC_50_ of protodioscin and dioscin compounds. After 48 h of incubation, cells were crushed in 0.1% trichloroacetic acid (TCA). The homogenate was centrifuged at 12,000 g for 15 min at 4°C, and the layer was kept in the dark for 1h after mixing with phosphate buffer (10 mM, pH 7.0) and potassium iodide (1 M). The absorbance of the resulting solution was measured at 390 nm. H_2_O_2_ concentrations were calculated using a standard curve.

### Malondialdehyde (MDA) content determination

Lipid peroxidation measured as Malondialdehyde content in MDA-MB-468 cells was determined using thiobarbituric acid (TBA) according to the method described previously [19] with minor modifications. In brief, cell homogenate, in different conditions described above, was mixed with trichloroacetic acid (20%) and TBA (0.67%). The mixture was heated at 95°C for 1h. After cooling, 1 mL *n*-butanol was added to the mixture followed by centrifugation at 12,000 g for 10 min. Organic supernatant was collected to measure the absorbance at 532 nm.

### Enzyme activity assays

#### Preparation of cell extracts for antioxidant enzyme assays

MDA-MB-468 and MCF-7 tumor cells were treated with protodioscin or dioscin compounds for 24 and 48 hours, respectively. Then, after washing once with PBS (10 mM, pH 7.4), the cells were harvested and centrifuged at 1,200 g for 10 min. The pellet was suspended in 500 μL of lysis buffer composed of 50 mM Tris-HCl, 1mM phenylmethanesulfonyl (PMSF), 0.1% (*v/v*) Triton X-100, in 1.5 mL Eppendorf tubes and maintained in constant agitation at 4°C for 30 min. The homogenate was centrifuged (1,600 g, 20 min) at 4°C. The supernatant (enzyme extract solution) was kept at −80°C or used for the determination of Superoxide dismutase (SOD), Glutathione Peroxidase (GPx), Thioredoxin reductase (TrxR), glutathione reductase (GR), and Isocitrate dehydrogenase (NADP+-ICDH) activities.

#### Antioxidant enzyme assays

The total SOD activity was assayed according to the method of Sun et al. [20] with some modifications. Briefly, the reaction mixture was composed of 0.05 M phosphate buffer, pH 7.5, 10 mM methionine, 0.1 μM EDTA, 2 μM riboflavin, 75 μM Nitro Blue Tetrazolium (NBT), and the enzyme extract. The SOD activity was measured at 560 nm. One unit of SOD activity was defined as the quantity of SOD required to obtain a 50% inhibition of the reduction of NBT. The activity was expressed as units per mg of protein content.

The total GPx activity was measured by the method of Lawrence and Burk [21] with some modifications. The reaction mixture contained 0.1 M potassium phosphate, pH 7.0, 1 mM EDTA, 1mM sodium azide, 1 mM GSH, GR (10 μg/mL), 0.25 mM NADPH and enzyme extract. The mixture was incubated at 25 ˚C for 3 min and completed by adding 0.25 mM of H2O2. The rate of NADPH oxidation was monitored at 340 nm for 5 min. GPx activity was calculated and expressed as nmol of NADPH oxidized/min/mg protein by using the extinction coefficient of 6.2 mM^–1^ cm^-1^.

The total TrxR was measured as the reduction of DTNB (5,5’-dithiobis (2-nitrobenzoic acid)) in the presence of NADPH [22]. The reaction mixture contained 0.1 M phosphate buffer, pH 7.6, 1 mM EDTA, 0.25 mM NADPH, 1 mM DTNB and enzyme extract. The increase in the absorbance at 412 nm was monitored at 25°C. TrxR activity was expressed as nmol of DTNB reduced/min/mg protein by using the extinction coefficient of 13.6 mM^–1^cm^–1^.

The total GR activity was estimated by a modified method of Carlberg and Mannervik [23]. Briefly, the reaction mixture contained 0.1 M phosphate buffer, pH 7.6, 1 mM GSSG, 0.2 mM NADPH. The contents were incubated at 25°C for 3 min, and the reaction was initiated by adding enzyme extract. The rate of NADPH oxidation was monitored at 340 nm. GR activity was expressed as nmol of NADPH oxidized/min/mg protein by using the extinction coefficient of 6.2 mM^–1^ cm^–1^.

NADP+-ICDH activity was estimated according to the procedure of Leterrier et al. [24].

#### Protein content determination

The total protein content of the samples was determined following the method of Bradford [25] using BSA as a protein standard.

### Statistical approaches

Data were subjected to one-way ANOVA, and differences were determined by Tukey’s multiple comparison test with GraphPad Prism 9.0.2 (134) software for macOS. Each experiment was repeated at least three times. Data are the means of individual experiments and presented as mean ± standard deviation (SD); p <0.05 was considered statistically significant.

## Results and discussion

### Cytotoxic effects of protodioscin and dioscin-treated human breast cancer cells

**Fig 2:**
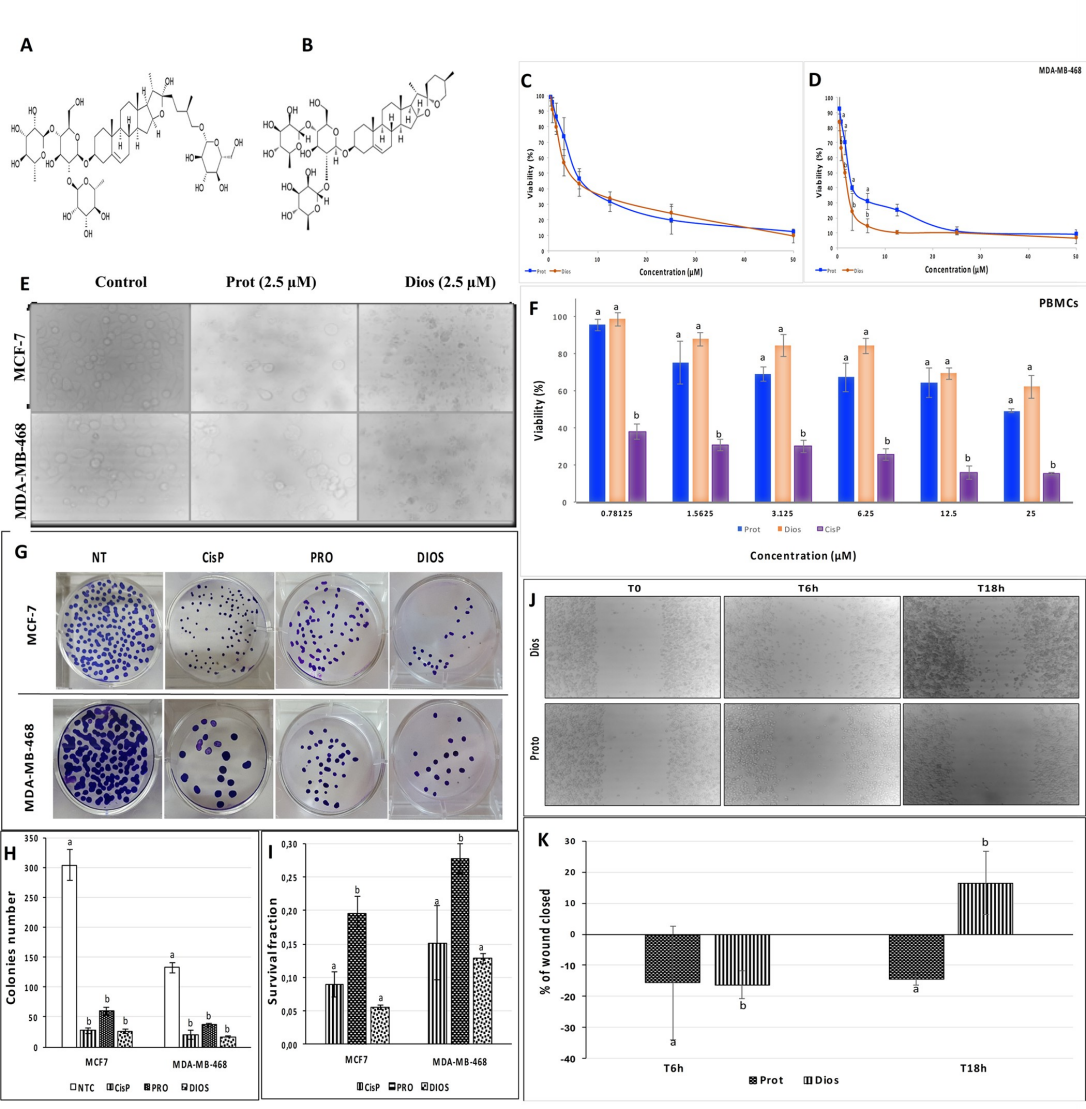
Effect of protodioscin and dioscin on cell growth, clonogenic formation and migration of non-invasive and invasive human breast cancer cell lines (MCF-7 and MDA-MB-468). (**A** and **B**) *In vitro* antitumor activity of protodioscin and dioscin towards adenocarcinoma cell line ER+ (MCF-7) and ER- (MDA-MB-468) after 48 hours of treatment. (**C**) *In vitro* cell proliferation morphological changes were observed after treatment for 48 hours (**×** 200, cells all seeded at the same initial density). (**D**) Viability of PBMCs treated with protodioscin or dioscin for 48 hours. (**E**, **F** and **G**) (**E**) Sample plate images from the clonogenic assay for cells treated with 1 μM of protodioscin/dioscin or CisP (0.33 M) for 10 days; (**F**) total number of colonies formed; (**G**) survival fraction. (**H** and **I**) Migration assay performed on MDA-MB-468 cells treated with 1 μM of protodioscin/dioscin or CisP (0.33 M) for 6 and 18 hours. Data represented as the mean of three independents measurements. Bars represent the standard error. Different letters indicate significant differences among treatments. *P-value* <0.05 indicate significant difference.

Accumulated evidence has demonstrated the potent anti-cancer activity of protodioscin and dioscin in various types of human cancer cell lines [26,27]. In agreement with these findings and in addition, the presebt study showed that both protodioscin and dioscin considerably suppressed cell proliferation in both breast cancer cells, ER-positive (MCF-7) and ER-negative (MDA-MB-468) (Fig 2A and 2B) in a dose-dependent manner as shown in the cell viability assay.

Furthermore, the cytotoxic effect was higher in ER-negative treated cells compared to ER-positive treated cells. The cell viability revealed that the half-maximal inhibitory concentrations (IC_50_) of dioscin were 1.53 μM in MDA-MB-468 and 4.79 μM MCF-7; and 2.56 μM and 6 μM for protodioscin in MDA-MB-468 and MCF-7 cells, respectively (Table 1).

**Table 1:** The in vitro antitumor IC_50_ values of protodioscin or dioscin against adenocarcinoma tumor cells (MDA-MB-468, MCF-7) and normal cells (PBMCs). Cisplatin was used as a drug reference.

Compound	IC_50_ values (μM) against adenocarcinoma tumor and normal cells		
	MDA-MB-468	MCF-7	PBMCs
*Prot*	2.56±0.38^a^	6±1.27^a^	>80^a^
*Dios*	1.53±0.17^b^	4.79±1.38^a^	= 40^a^
CisP	0.15±0.016^c^	2.16±0.35^b^	<1^b^

Data represented as mean ± standard deviation of three independent replicates. Different letters in the same column indicate significant differences (*p* <0.05) within conditions according to the two-way ANOVA multiple comparison range Test.

Microscopic observation showed a substantial effect on the cell morphology followed this significant cell death after 48 h of treatment with protodioscin and dioscin (Fig 2E). The cells treated with protodioscin or dioscin exhibited cytoplasmic shrinkage and began to detach from each other and become less plate adherent compared to the untreated cells. Our observations confirmed the previous malformation of the cell profile, condensation of the cytoplasm, plasma membrane blebbing, cell shrinkage, and nucleus condensation that appeared in dioscin-treated cells [16].

### Cytotoxicity against PBMCs as normal cells

Recall that PBMCs are the first normal cell populations that meet antitumor drugs used in conventional chemotherapy and collapse from the first week of intravenous treatment of patients, resulting in significant immune deficiency and increased side effects [16]. The primary intention of cancer chemotherapy is to target cancer cells without displaying toxicity toward normal cells specifically. In this work, the cytotoxicity on PBMCs proved that these normal cells were more tolerant to steroidal treatment than MCF-7 and MDA-MB-468 cell lines, as represented in Fig 2D. Both steroidal compounds demonstrated weak cytotoxicity (*p* <0.05) on PBMCs cells with an IC_50_ ≥ 50 μM. According to the American National Cancer Institute (NCI), the criteria for a pure molecule to exhibit cytotoxic effect is an IC_50_ <30 μg/mL. This PBMCs tolerance proved a selective killing ability of these molecules towards both adenocarcinomas’ tumor, ER-positive, and TNBC breast cancer cells.

### Clonogenic assay of MDA-MB-468 and MCF-7 treated with protodioscin and dioscin

Colony formation is a marked parameter reflecting the extent of tumor malignancy, contributes to tumor recurrence [17], and is suspected of leading the therapeutics resistance. In this respect, to extend our observations on the cytotoxicity effect, the clonogenic survival potential of tumor cells was evaluated to determine long-term cell survival ability. Protodioscin and dioscin cell treatment resulted in a substantial decrease in the clonogenic capacity of both MCF-7 and MDA-MB-468 compared with the non-treated control (Fig 2E–2G). Interestingly, the adenocarcinoma MCF-7 cells were more sensitive than MDA-MB-468 (Fig 2E). Further, dioscin showed higher potent inhibitory for both cell lines in blocking colony formation. Furthermore, no significant differences were observed between the number of colonies survival cells (CSCs) in dioscin and CisP in both adenocarcinoma-treated cells (p > 0.05). In conclusion, the colony formation assay showed the anti-clonogenic survival potential of protodioscin and dioscin, in addition to inhibiting the proliferation and progression of breast cancer cells. Furthermore, our observation of breast cancer cell lines confirmed the report describing the capacity of dioscin to inhibit the clonogenic formation ability on B16 cells in a dose‐dependent manner [28] and on PC3 cells [29].

### The invasive breast cancer cell migration assay

The effect of dioscin and protodioscin on cell migration on triple-negative MDA-MB-468 cells was performed by a wound healing migration assay. As indicated in Fig 2H and 2I, compared to the negative control (DMSO treated cells), the induction of dioscin reduced cell migration up to 16.5% after 18 h. Moreover, our data also revealed that protodioscin was able to detach the invasive cell line immediately after treatment. Additionally, the migration inhibition was more significant in the invasive breast cancer cells (MDA‐MB‐468) treated by protodioscin.

### Effect of protodioscin and dioscin on oxidative stress

The deregulation of cellular energetics is one of the hallmarks of cancer cells. Because of the increased metabolic demands of sustained proliferation [30], cancer cells are under tremendous intrinsic oxidative stress associated with further elevation of ROS relative to non-cancer cells [31]. This elevation of ROS can promote tumor growth, malignant progression, invasion, and migration of various types of cancer, such as breast cancer [32]. However, extreme levels of ROS may be lethal for tumor cells themselves. Many studies have shown the induction of ROS-mediated apoptosis by various medicinal herbs in breast cancer cells. As a matter of fact, a large number of research studies have outlined the implication of elevated levels of H_2_O_2_ in the induction of apoptosis [10,33]. Specifically, growing evidence has demonstrated that low levels of H_2_O_2_ facilitate tissue regeneration and enhance the proliferation and migration of mammalian cells [34]. Accordingly, targeting ROS or oxidative stress is an important anticancer therapeutic strategy proposed in breast cancer therapy [10].

**Fig 3:**
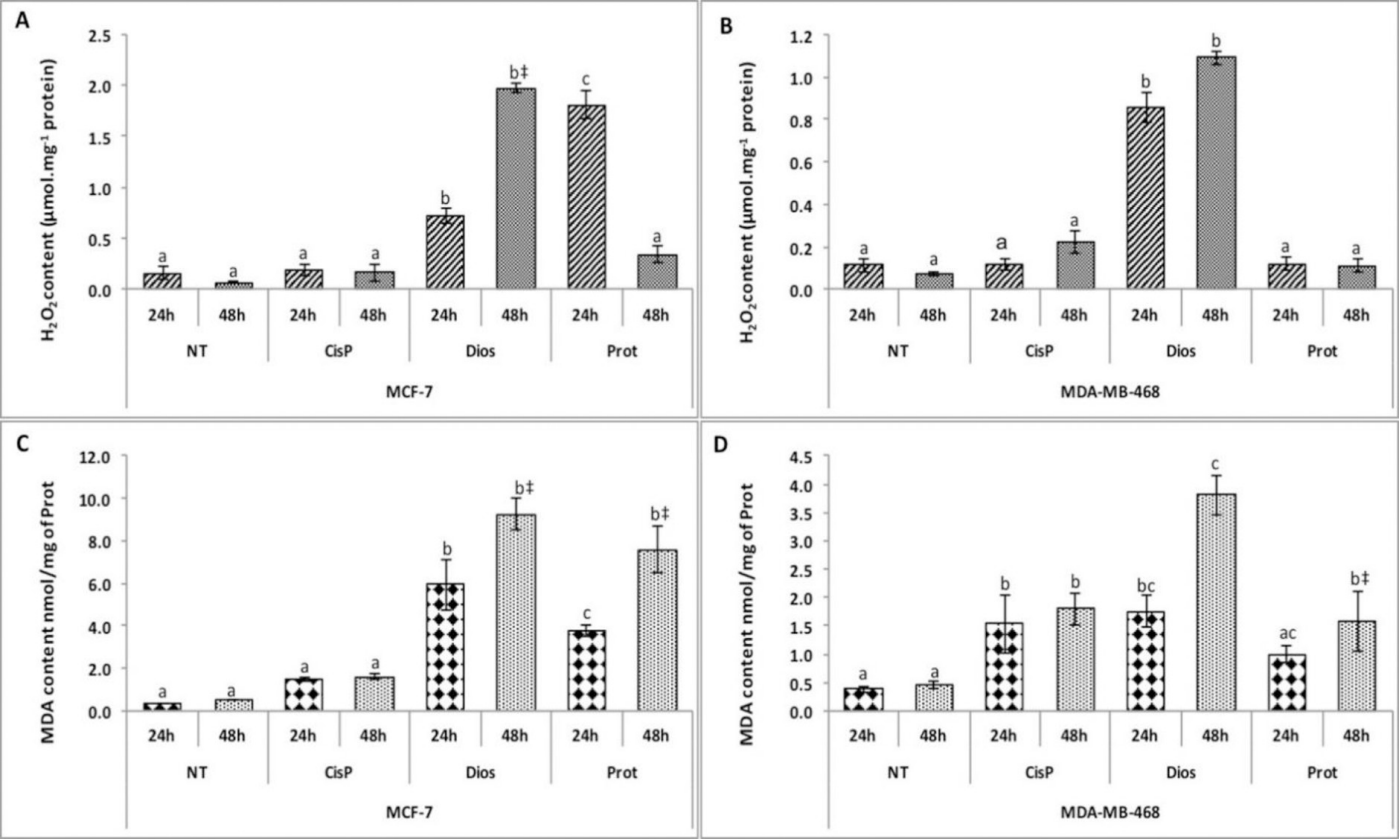
Protodioscin and dioscin increase ROS generation in the adenocarcinoma MCF-7 and MDA-MB-468 cell lines. (**A/B**) hydrogen peroxide (H_2_O_2_) content; (**C/D**) malondialdehyde (MDA) concentration. NT (not treated cells) and CisP (Positive control). Data are represented as the mean of six replicates issued from two independent experiments. Bars represent the standard error. Different letters indicate significant differences among treatments in the same tumor line at *p* <0.05. The signs *, †, and ‡ indicate a significant difference between the time treatment at *p* <0.05, *p* <0.01, and *p* <0.0001, respectively.

In this study, e it was demonstrated that both protodioscin (IC_50_) and dioscin (IC_50_) induced a significant increase of H_2_O_2_ content, as compared to the negative control, in both tumor lines for dioscin and MCF-7 for protodioscin (Fig 3A and 3B). These compounds have the capacity to increase more than four times the level of H_2_O_2_ contents in MCF-7 and MDA-MB-468 treated by dioscin and protodioscin (Fig 3A and 3B). In addition, lipid peroxidation, another indicator of oxidative stress, was quantified by measuring the MDA content (Fig 3C and 3D), showing that both steroidal saponins tested in this study induced a significant increase in the MDA content compared to the negative control. The increases in the MDA contents reached up to 8-fold and 18-fold in MDA-MB-468 (Fig 3D) and MCF-7 tumor cells (Fig 3C), respectively. However, the effect of dioscin was generally more pronounced compared to protodioscin. Moreover, MCF-7 treated cells showed higher MDA levels than the MDA-MB-468 cells.

Increased levels of H_2_O_2_ and MDA under both treatment conditions may be responsible for triggering oxidative stress in the breast cancer cell lines, resulting in instability of DNA, lipids, and proteins and leading to cell death, as reported in several previous works [35–38]. The increase in oxidative stress could be a consequence of an imbalance between antioxidants and prooxidants. To verify this hypothesis, activities of enzymes responsible for protecting cells from oxidative stress, including SOD, GPx, GR, and TrxR, were determined.

**Fig 4:**
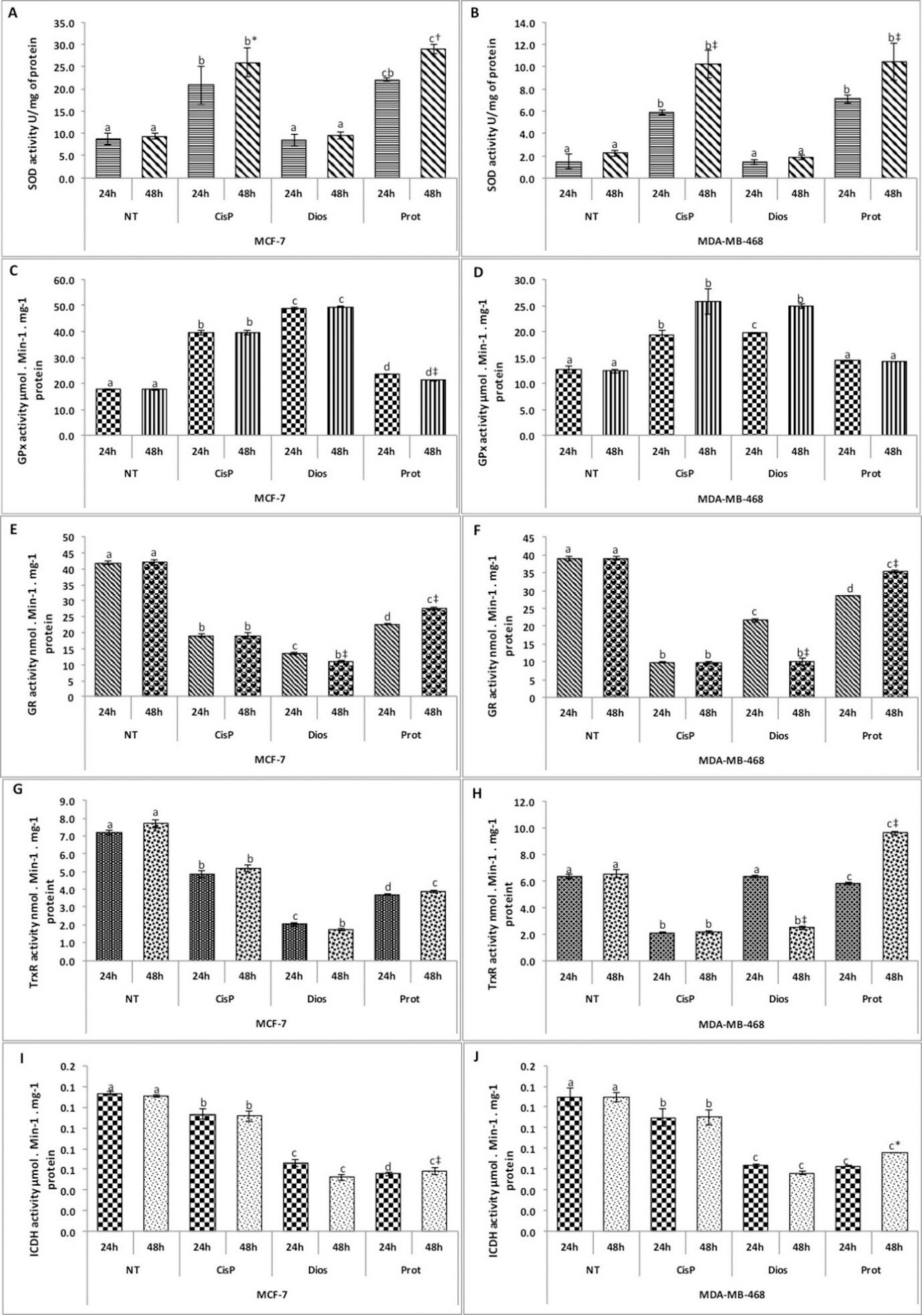
Effect of protodioscin and dioscin on the antioxidant defense system of the adenocarcinoma MCF-7 and MDA-MB-468 cell lines. **(A** and **B**) superoxide dismutase (SOD) enzymatic activity. (**C** and **D**) glutathione peroxidase (GPx) enzymatic activity. (**E** and **F**) glutathione reductase (GR) enzymatic activity. (**G** and **H**) thioredoxin reductase (TrxR) enzymatic activity. (**I** and **J**) Isocitrate dehydrogenase (ICDH) enzymatic activity. NT (Non-treated cells) and CisP (Positive control). Data are represented as the mean of six replicates issued from two independent experiments. Bars represent the standard error. Different letters indicate significant differences among treatments in the same cell line at *p* <0.05. The signs *, †, and ‡ indicate a significant difference between the time treatment at *p* <0.05, *p* <0.01, and *p* <0.0001, respectively.

The total antioxidant enzymes, including superoxide dismutase (SOD) and glutathione peroxidases (GPxs), have been reported to be upregulated in cancer cells to prevent oxidative damage and enable cancer cell survival under oxidative stress. Superoxide dismutase is recognized as the primary defense barrier against ROS, which catalyzes the dismutation of superoxide anion radicals (O_2_^.^) into hydrogen peroxide (H_2_O_2_). H_2_O_2_ is then eliminated by its conversion to H_2_O by Catalase (CAT) or GPx. The present study showed that both steroidal protodioscin (IC_50_) or dioscin (IC_50_) increased significantly in at least one of the phase I antioxidant enzymes (SOD or GPx) in both cell lines (Fig 4A and 4B). Protodioscin increased SOD activities in both cell lines. Likewise, dioscin increased GPx activities in both cell lines, while protodioscin only minimally induced GPx in MCF-7 cells. For the GPx enzyme, this increase reached its maximum in the MCF-7 tumor cells treated with the dioscin after 48 h. For SOD enzyme activity, this increase reached its maximum in the MDA-MB-468 cells treated with protodioscin after 24 h. Increased antioxidant enzyme activities might be one of the strategies used by cancer cells to cope with the oxidative stress caused by both steroidal saponins tested here [6]. However, protodioscin and dioscin increase the oxidative stress alterations to levels that the tumor cells failed to support. In fact, as described below, the significant increase of the MDA content after 48 h of treatment indicated that even with the increase of the antioxidant enzymes, SOD and GPx could not stop the oxidative stress damage.

Several reports have indicated the ability of dioscin to induce ROS generation leading to cell death. Indeed, Zhiyu et al. (2012) have shown an increase in oxidative stress and apoptosis in human Kyse-510 esophageal cancer cells after treatment with dioscin [39]. Dioscin was also reported to induce a promising inhibiting activity of glioblastoma cells through an increase in ROS generation and DNA damage [40]. In a recent study, Yao et al. [41] noted an increase in the ROS production after treatment with dioscin in lung squamous cell carcinoma cells, which results in the activation of the apoptosis pathways. Regarding protodioscin, few studies have reported its effect on cancer cells. One of these studies was conducted by Lin et al. [42] who showed that protodioscin could induce apoptosis in human cervical cancer cells via ROS-mediated endoplasmic reticulum stress.

To maintain ROS to their appropriate levels in the cells, two antioxidant systems are implicated: glutathione- and thioredoxin-dependent systems. Glutathione (GSH) and thioredoxin are major sources of reducing equivalents required for the key enzymes involved in regulating redox homeostasis, such as peroxidases and thiol reductases [43]. Thus, to help understand the possible reason for the ROS increase to levels that cells were not able to support, the effect of protodioscin and dioscin on TrxR and GR was evaluated.

Fig 4E and 4F report the effect of the exposure to protodioscin (IC_50_) and dioscin (IC_50_) on GR and TrxR activities of MDA-MB-468 and MCF-7 tumor cells. The effect depended on the tested compound and the cell line used. Indeed, the GR activity decreased after cell exposure to both compounds; however, the effect of dioscin was higher. GR activity decreased by 74% and 73% in MDA-MB-468 and MCF-7 cells after treatment with dioscin for 48 h. The effect of these compounds also affected the TrxR activity. Indeed, this activity decreased by 49% and 77% in MCF-7 exposed to protodioscin and dioscin, respectively. As for the MDA-MB-468 cell line, TrxR activity decreased 62% after 48 h exposure to dioscin compounds, respectively (Fig 4G and 4H).

GSH has been found to be elevated in many tumor cells that are resistant to cancer therapy. Recently, Keleher et al. [44] indicated that the depletion of GSH and the inhibition of TrxR activity increased responses in human breast cancer MDA-MB-231 and MCF-7 cancer cells. Consequently, the results presented in this work suggest that both steroidal compounds may be considered valuable in vitro antitumor compounds that act through increasing intracellular ROS contents. Furthermore, these results indicate that both steroids’ in vitro antitumor activity could be due to the inhibition of GR and TrxR activities.It was reported that NADPH is an essential cofactor for the activity of the glutathione- and thioredoxin-dependent systems [45]. This cofactor is synthesized in the cell through different metabolic pathways, and one of the major enzymes implicated in the furniture of NADPH is NADP^+^-ICDH [34]. Moreno-Sánchez et al. [46] argued that decreased NADP^+^-ICDH expression leads to low resistance to oxidative stress, and the over-expression of NADP^+^-ICDH leads to high resistance [46]. In this study, exposure of ER-negative and ER-positive tumor cells to the (IC_50_) steroids protodioscin and dioscin significantly reduced the ICDH activity, mainly after 48 h treatment (Fig 4I and 4J). This is in line with our recent findings [6], showing that the decrease in the ICDH activity may be responsible for the depletion of NADPH, used to cope with oxidative stress through the glutathione- and thioredoxin-dependent systems.

## Conclusions

In conclusion, both steroidal compounds tested in this study, protodioscin, and dioscin, inhibited the proliferation of the human breast cancer ER-positive (MCF-7) and triple-negative breast cancer (MDA-MB-468) in a dose-dependent manner. In addition, protodioscin and dioscin blocked the clonogenic formation and the migration of the non-invasive and invasive cell lines.

Moreover, treatment of both subtypes of cancer cells with protodioscin and dioscin resulted in an increase in ROS generation, which indicated that this pathway could be responsible for the *in vitro* anti-tumor activity of these steroidal compounds. It was argued in several reports that the implication of ROS in the activation of different signaling pathways leads to cell death. For instance, in the study conducted by Kim et al. [47], it has been shown that ROS activates the GRP78/PERK signal transduction, which triggers apoptosis via endoplasmic reticulum stress in cervical cancer cells. Moreover, in another study, luteolin, a natural compound, was used to treat glioblastoma cells. According to this study, the effect was mediated by the endoplasmic reticulum stress response and mitochondrial dysfunction [48] Finally, in a recent report, Lin et al. [49] showed that protodioscin could be responsible for endoplasmic reticulum stress-dependent apoptosis in human cervical cancer cells, likely through the stimulation of the JNK and p38 pathways. Moreover, in another study, the authors tested the effect of alantolactone, a sesquiterpene lactone, and showed potent effects against the breast cancer cells MDA-MB-231 [50]. According to these authors, the effect was mediated by ROS production via the implication of the MAPK pathway.

For all the above results, protodioscin and dioscin may serve as promising new natural compounds for treating of TNBC and ER-positive breast cancer with minimal side effects. In addition, they could also serve as effective tools for the study of cell death through ROS-mediated signaling pathways. Therefore, an additional investigation should be considered to decipher these two compounds’ complete mechanisms of action and improve further their pharmacological properties and anticancer efficacy. Also, testing the two steroidal compounds in animal models (such as xenografted nude mice bearing MCF-7 and MDA-MB-468 tumors) is needed to confirm tumor reductions in an *in vivo* context.

## Supporting information

S1 Raw data (ZIP)
