# Supplementary material for: In vitro evaluation of dioscin and protodioscin against ER-positive and triple-negative breast cancer
Source: PLoS One. 2023 Feb 9;18(2):e0272781. doi: 10.1371/journal.pone.0272781 (PMC9910703; doi:10.1371/journal.pone.0272781)
Supplement: S1 Raw data — (ZIP) [file pone.0272781.s001.zip › Raw Data_Bouchmaa et al/Migration assay/Migration assay-revision-dios and proto/FIgure Migration-proto & Dios_finale.pptx]

## Slide 1
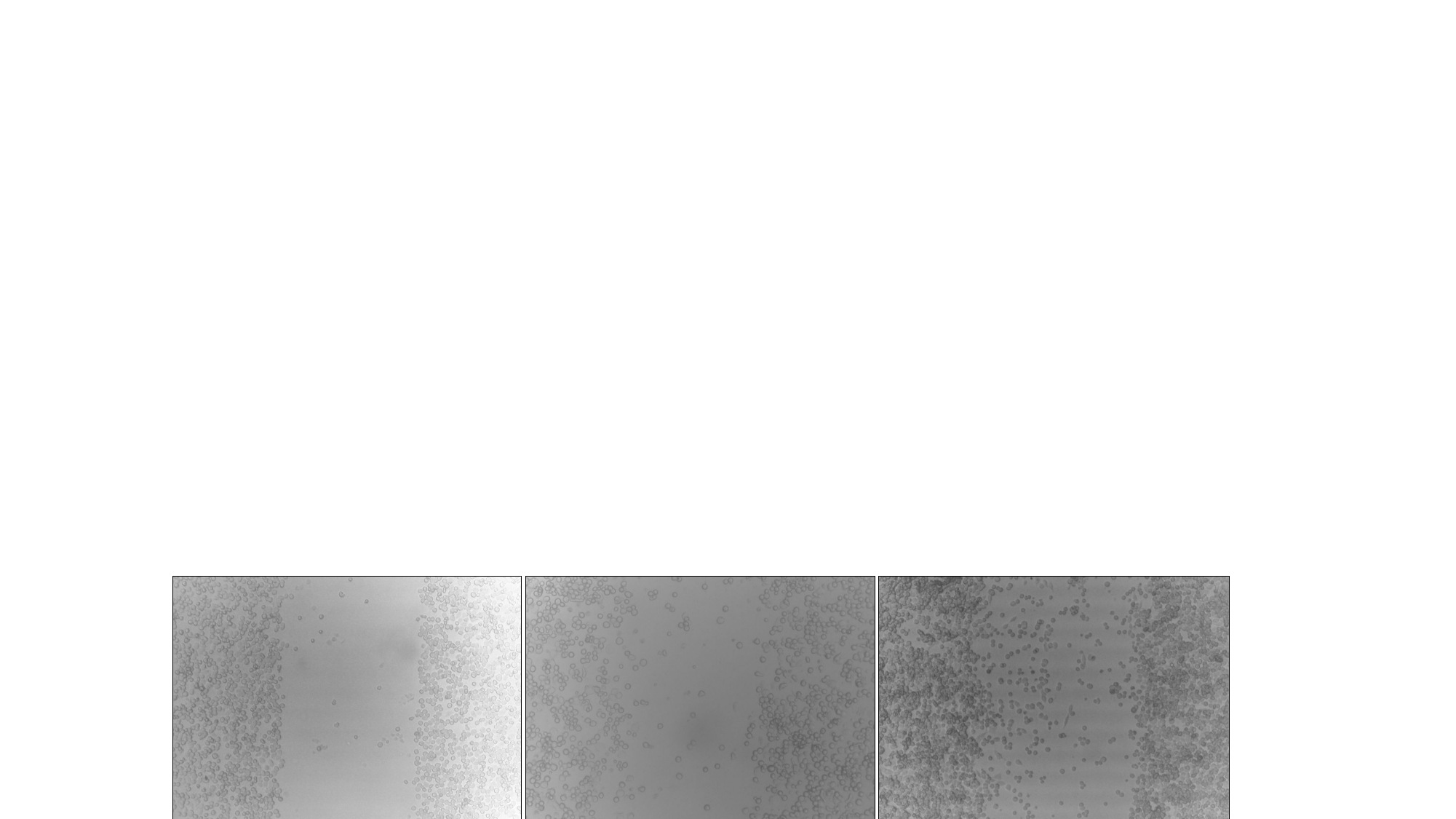

## Slide 2
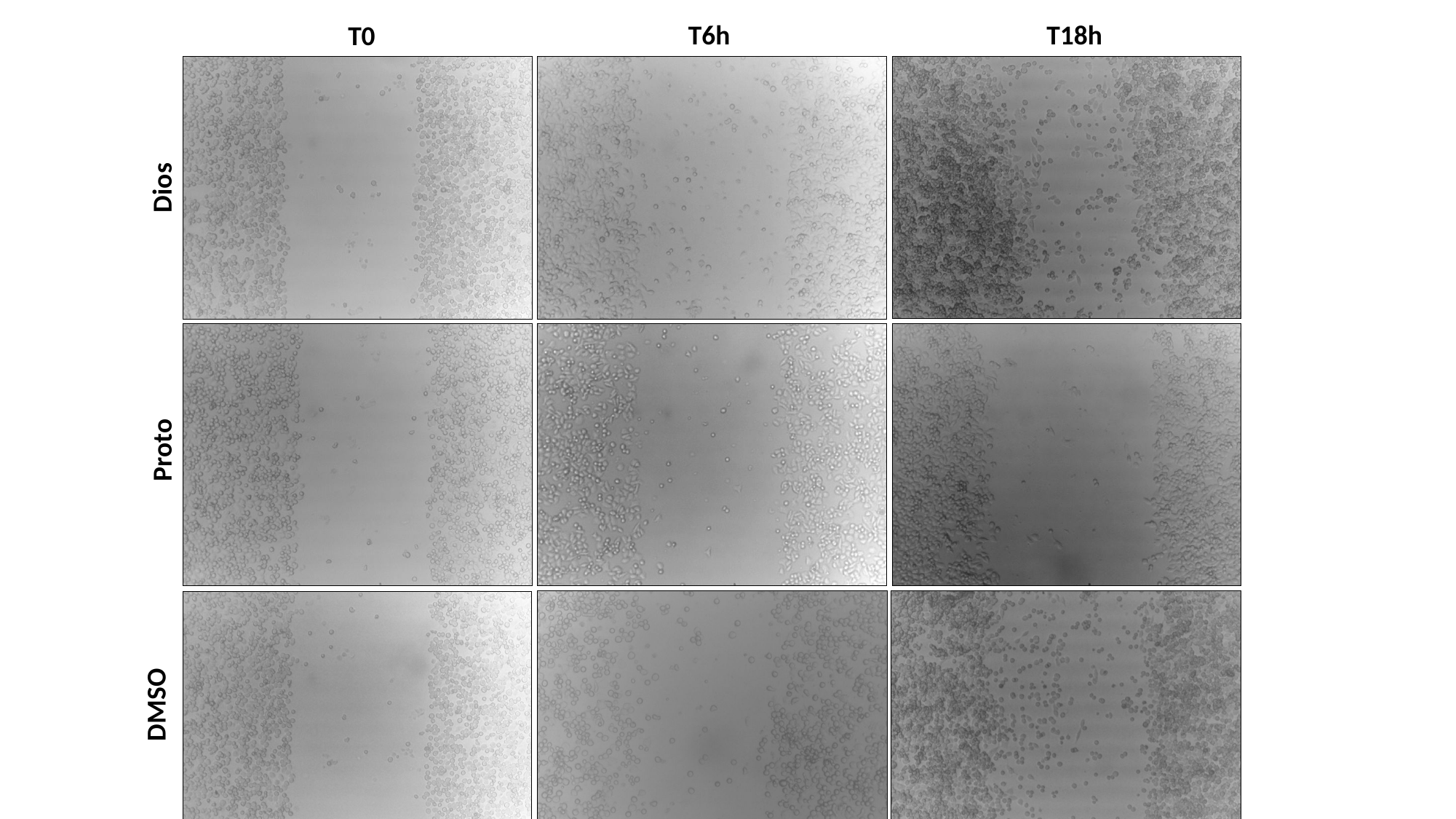

T6h
T18h
T0
Dios
T18h
Proto
DMSO

## Slide 3
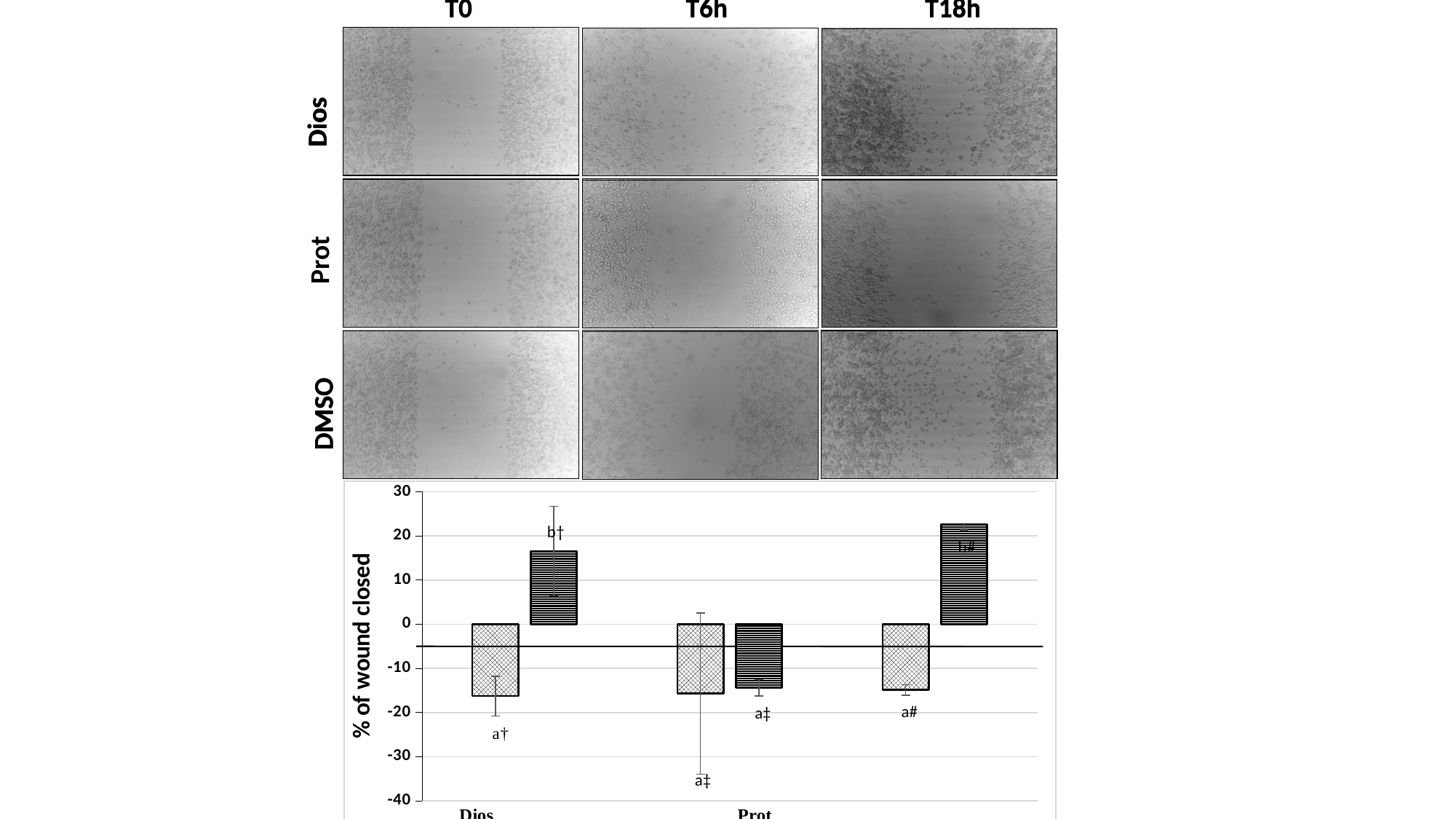

T0
T6h
T18h
Dios
T18h
DMSO
### Chart
| Category | T6h | T18h |
|---|---|---|
| Dios | -16.26 | 16.56 |
| Prot | -15.69 | -14.39 |
| DMSO | -14.86 | 22.63 |T0
T6h
T18h
Dios
T18h
Prot
DMSO
### Chart
| Category | T6h | T18h |
|---|---|---|
| Dios | -16.26 | 16.56 |
| Prot | -15.69 | -14.39 |
| DMSO | -14.86 | 22.63 |
