# Supplementary material for: In vitro evaluation of dioscin and protodioscin against ER-positive and triple-negative breast cancer
Source: PLoS One. 2023 Feb 9;18(2):e0272781. doi: 10.1371/journal.pone.0272781 (PMC9910703; doi:10.1371/journal.pone.0272781)
Supplement: S1 Raw data — (ZIP) [file pone.0272781.s001.zip › Raw Data_Bouchmaa et al/Migration assay/Migration assay.pptx]

## Slide 1
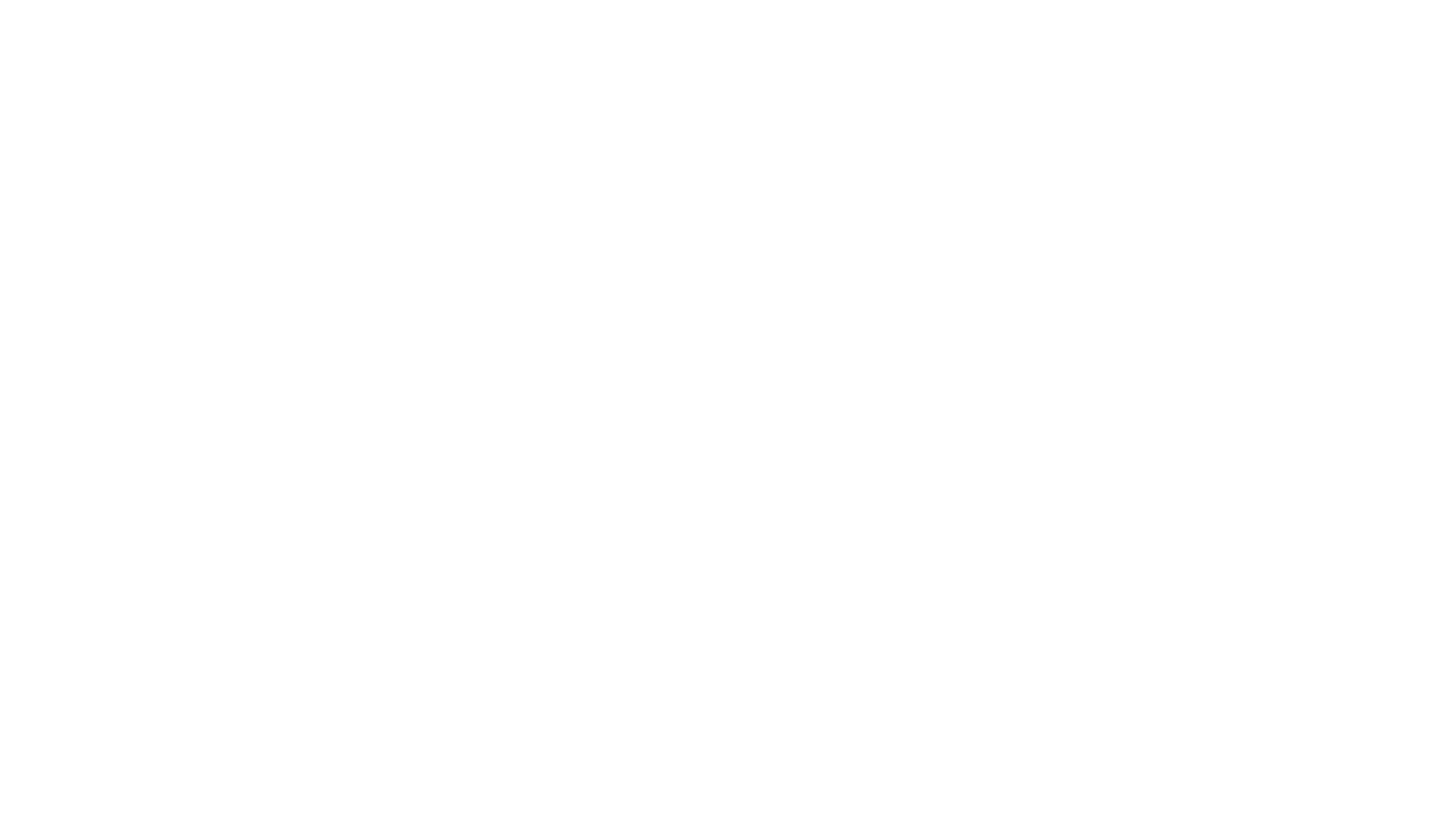

#

## Slide 2
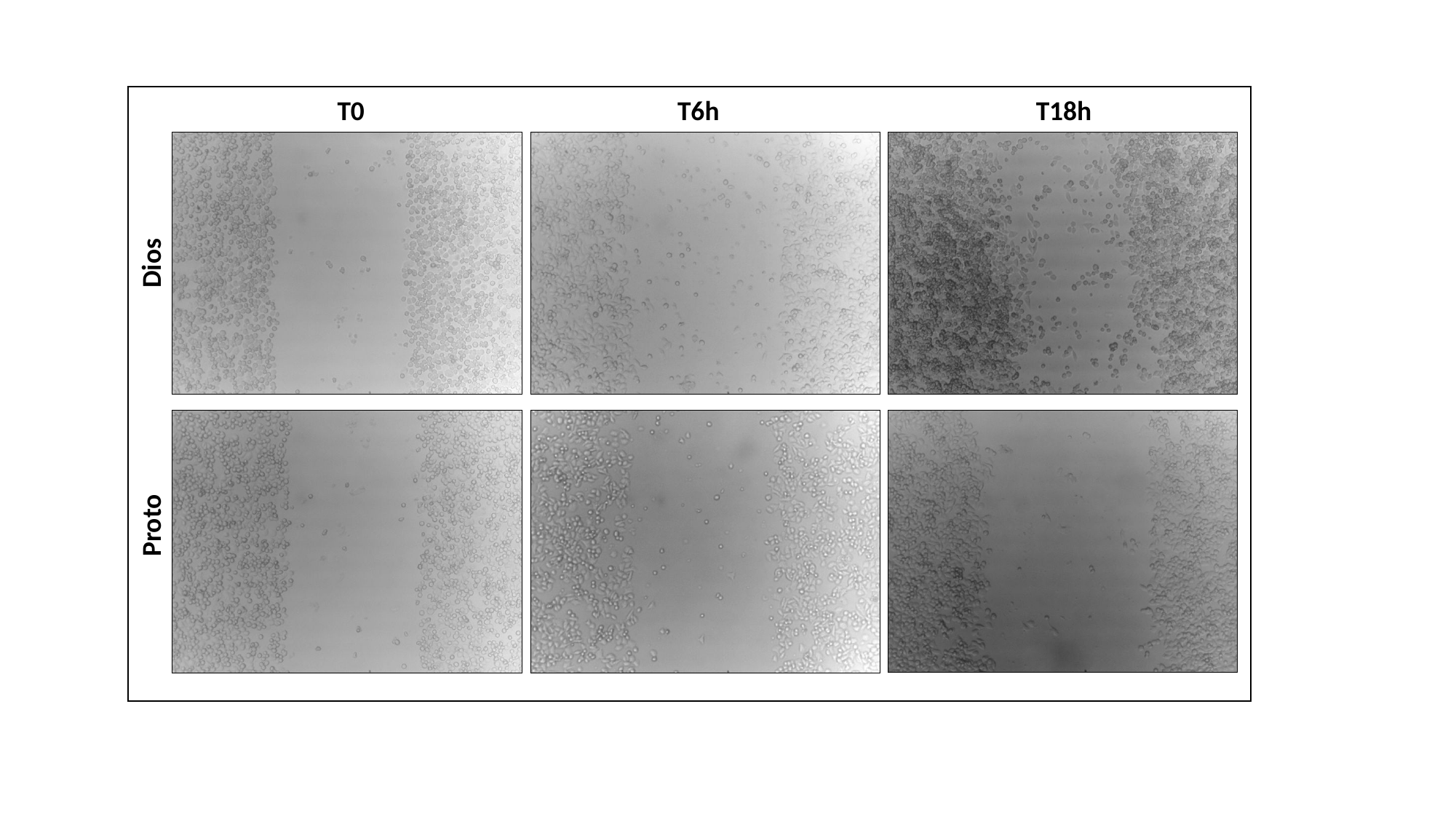

T6h
T18h
T0
Dios
T18h
Proto

## Slide 3
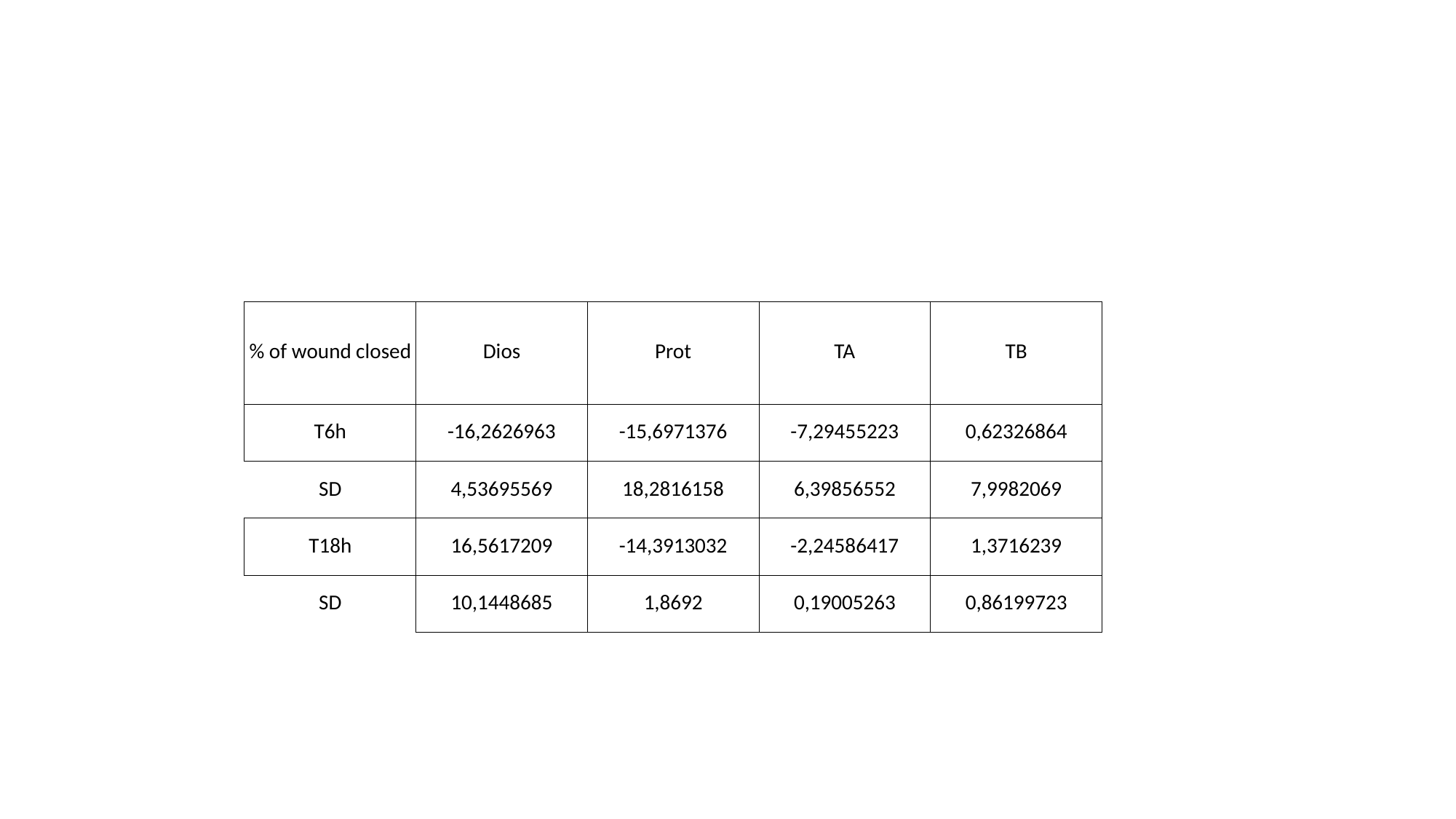

#
| % of wound closed | Dios | Prot | TA | TB |
| --- | --- | --- | --- | --- |
| T6h | -16,2626963 | -15,6971376 | -7,29455223 | 0,62326864 |
| SD | 4,53695569 | 18,2816158 | 6,39856552 | 7,9982069 |
| T18h | 16,5617209 | -14,3913032 | -2,24586417 | 1,3716239 |
| SD | 10,1448685 | 1,8692 | 0,19005263 | 0,86199723 |

## Slide 4
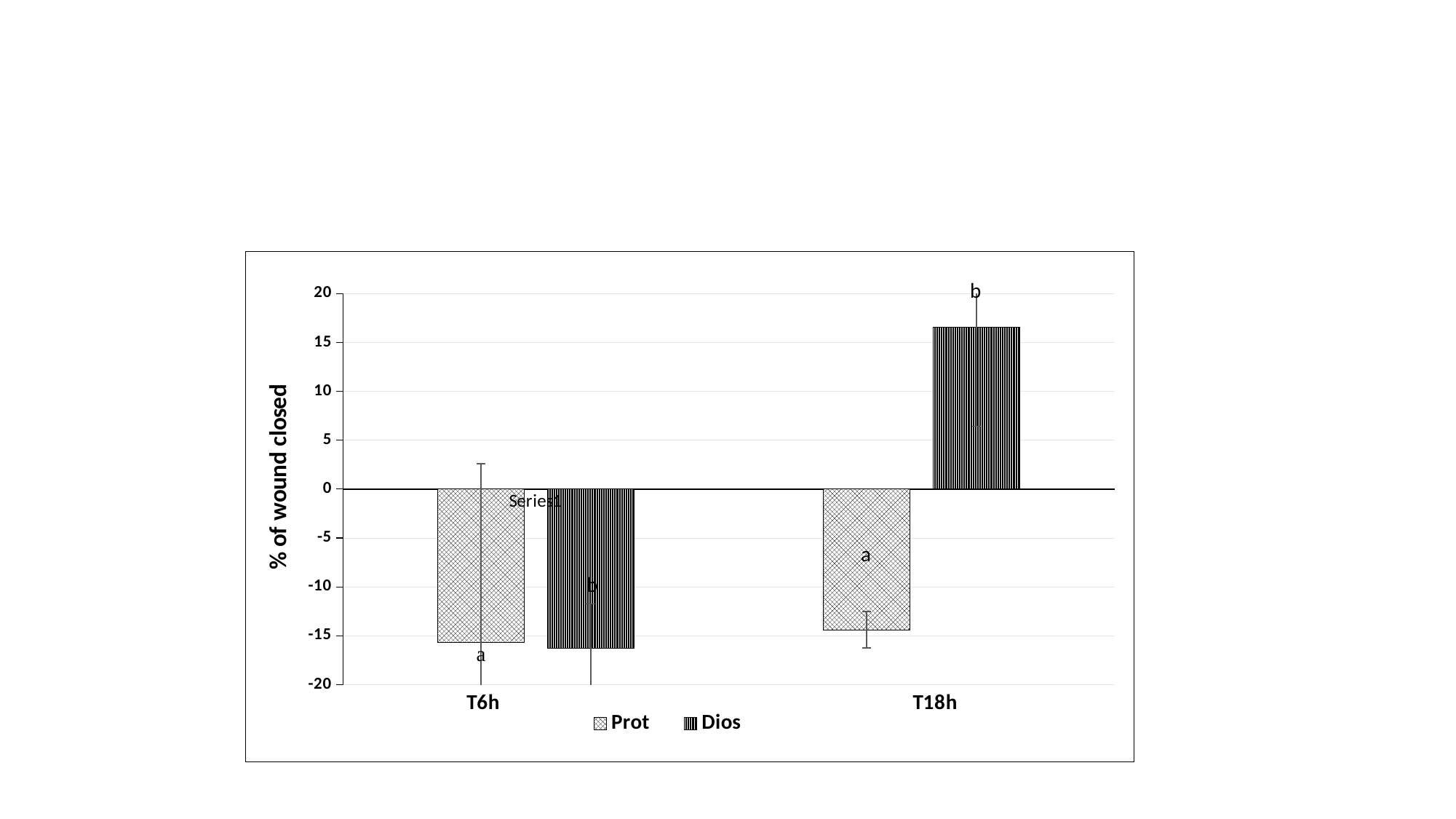

### Chart
| Category | Prot | Dios |
|---|---|---|
| | -15.69713764756299 | -16.26269628131503 |
| | -14.39130315927476 | 16.56172086597513 |
