# Supplementary figures and images for: In vitro evaluation of dioscin and protodioscin against ER-positive and triple-negative breast cancer
Source: PLoS One. 2023 Feb 9;18(2):e0272781. doi: 10.1371/journal.pone.0272781 (PMC9910703; doi:10.1371/journal.pone.0272781)

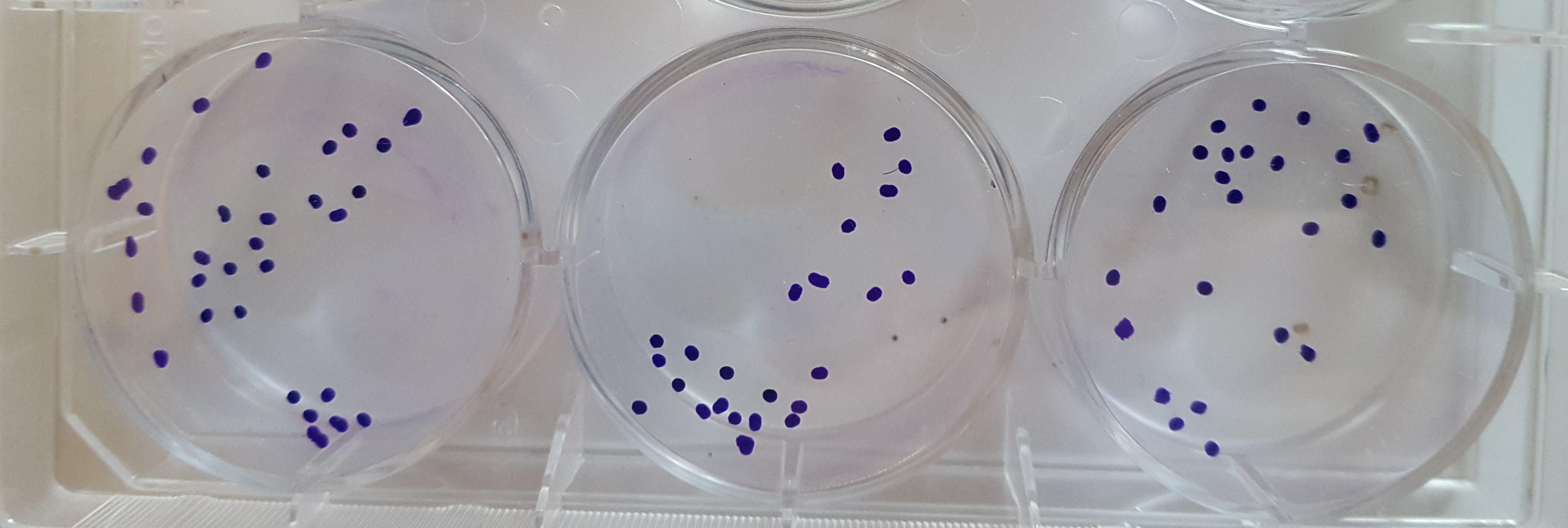

Supplement: S1 Raw data — (ZIP) [file pone.0272781.s001.zip › Raw Data_Bouchmaa et al/Clono/MCF7 (DIOS).tif]

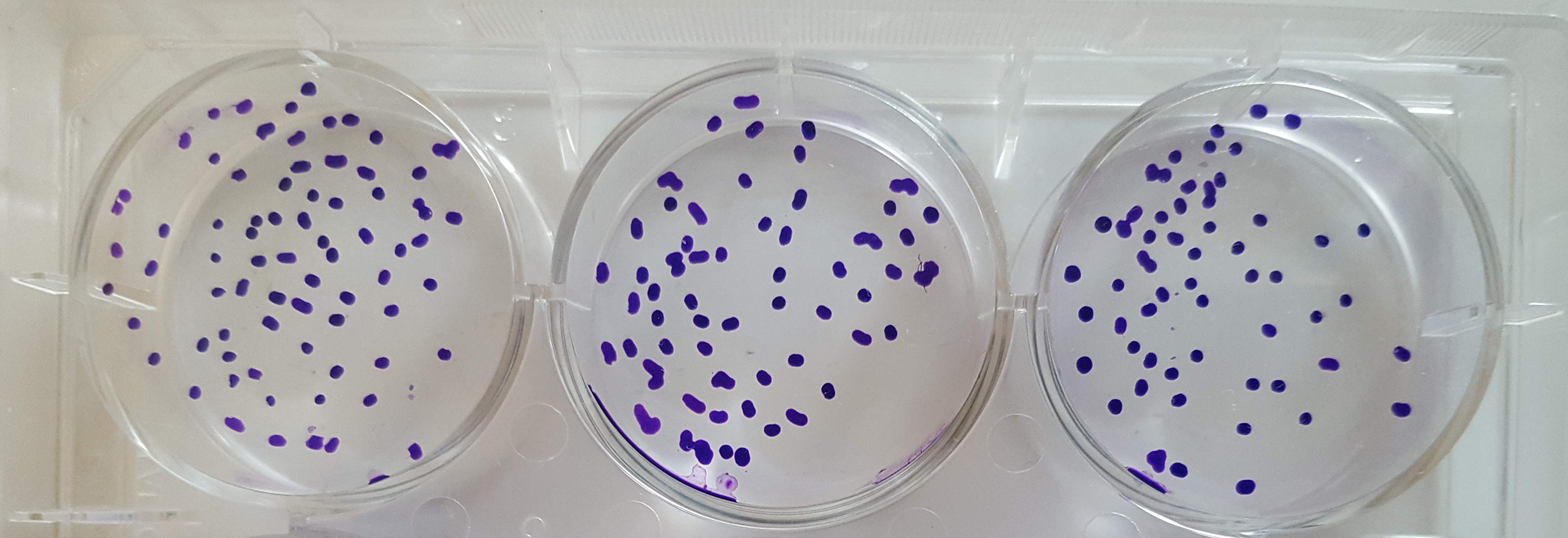

Supplement: S1 Raw data — (ZIP) [file pone.0272781.s001.zip › Raw Data_Bouchmaa et al/Clono/MCF7 (PRO).tif]

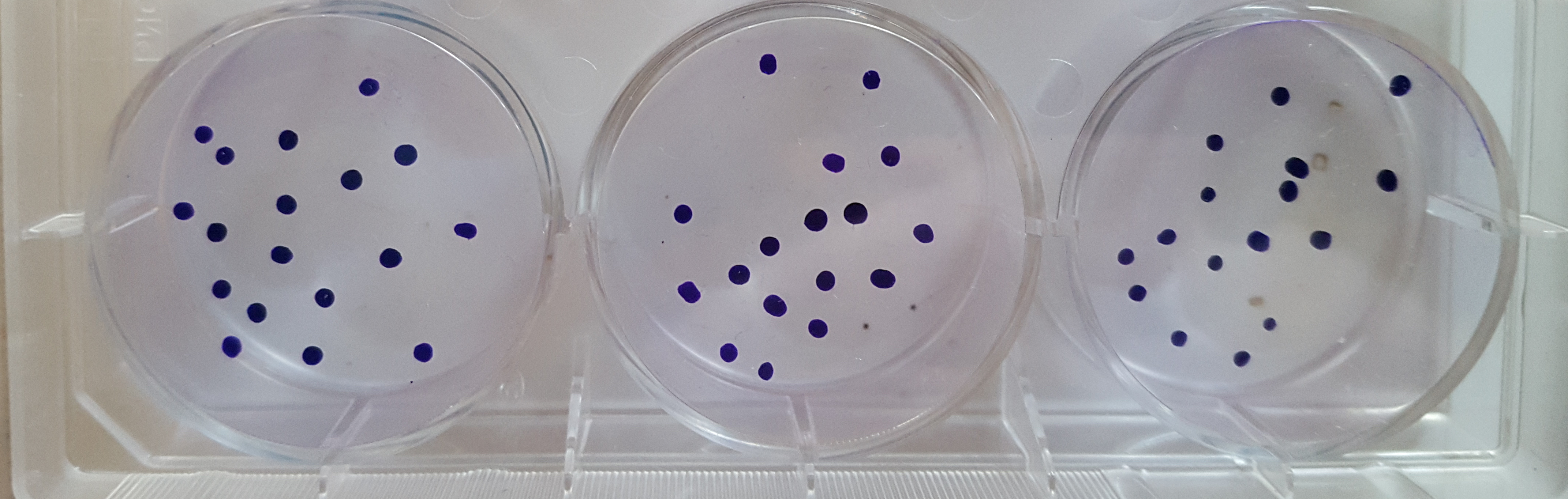

Supplement: S1 Raw data — (ZIP) [file pone.0272781.s001.zip › Raw Data_Bouchmaa et al/Clono/MDA (DIOS).tif]

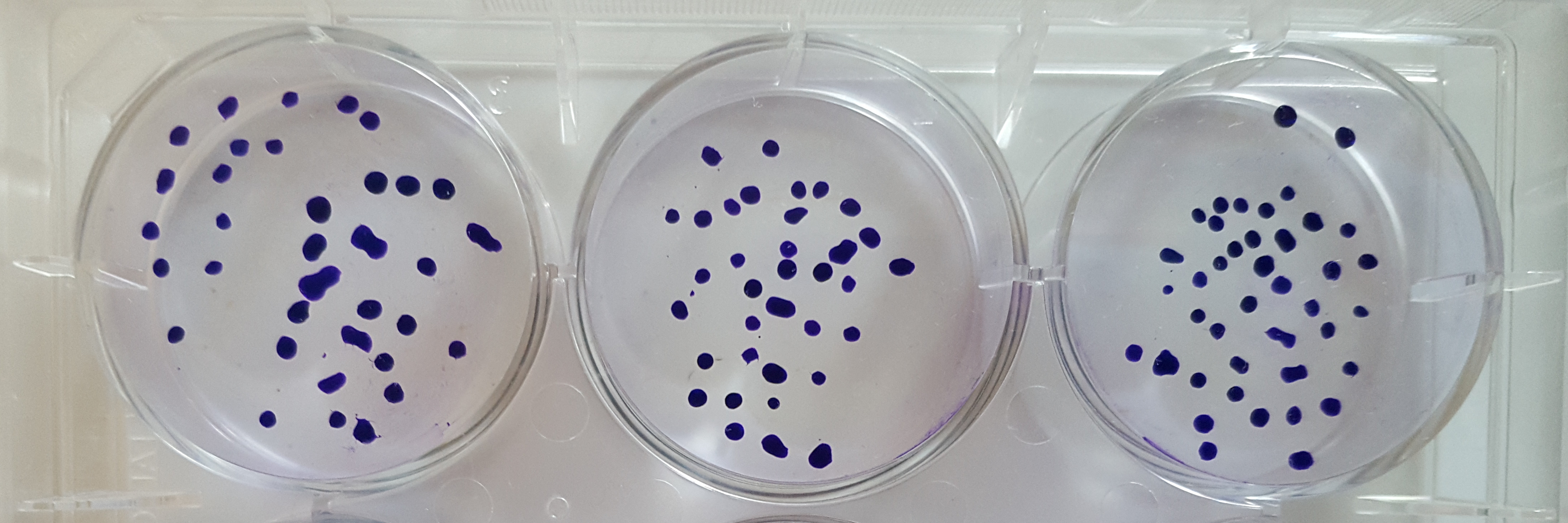

Supplement: S1 Raw data — (ZIP) [file pone.0272781.s001.zip › Raw Data_Bouchmaa et al/Clono/MDA (PRO).tif]

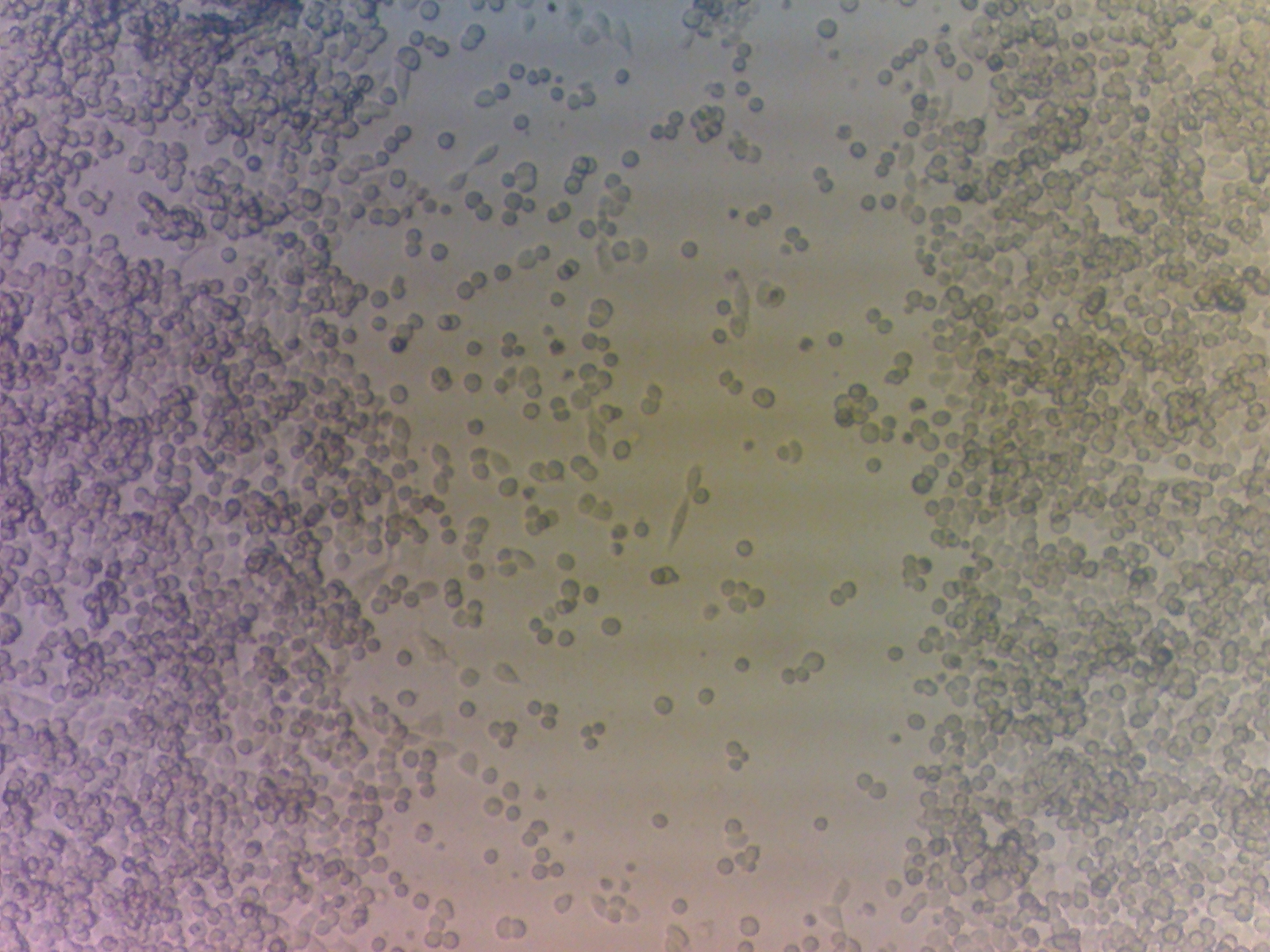

Supplement: S1 Raw data — (ZIP) [file pone.0272781.s001.zip › Raw Data_Bouchmaa et al/Migration assay/Migration assay-revision-dios and proto/Cneg/Cneg 18h MDA rep2.jpg]

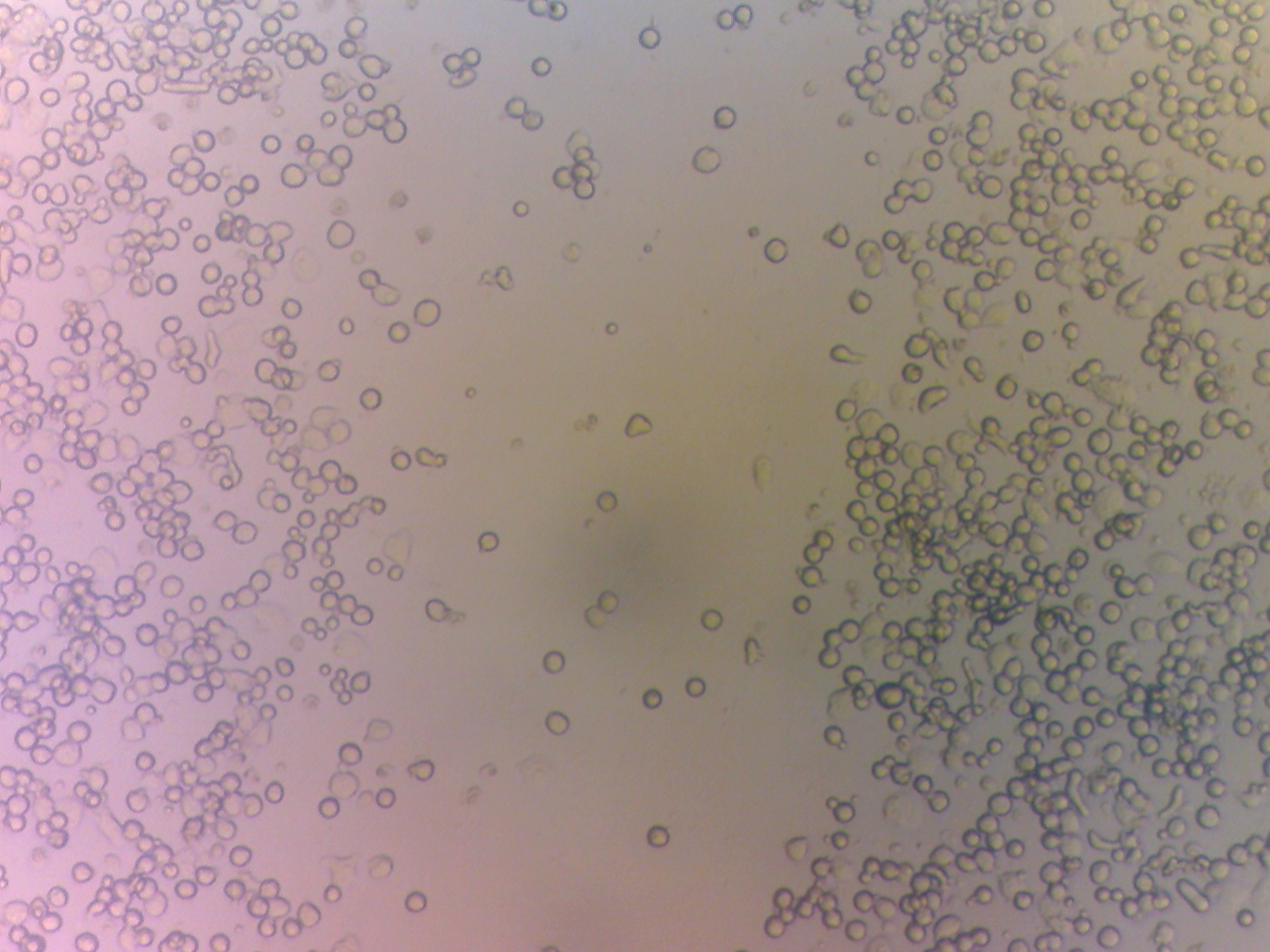

Supplement: S1 Raw data — (ZIP) [file pone.0272781.s001.zip › Raw Data_Bouchmaa et al/Migration assay/Migration assay-revision-dios and proto/Cneg/Cneg6h MDA rep2.jpg]

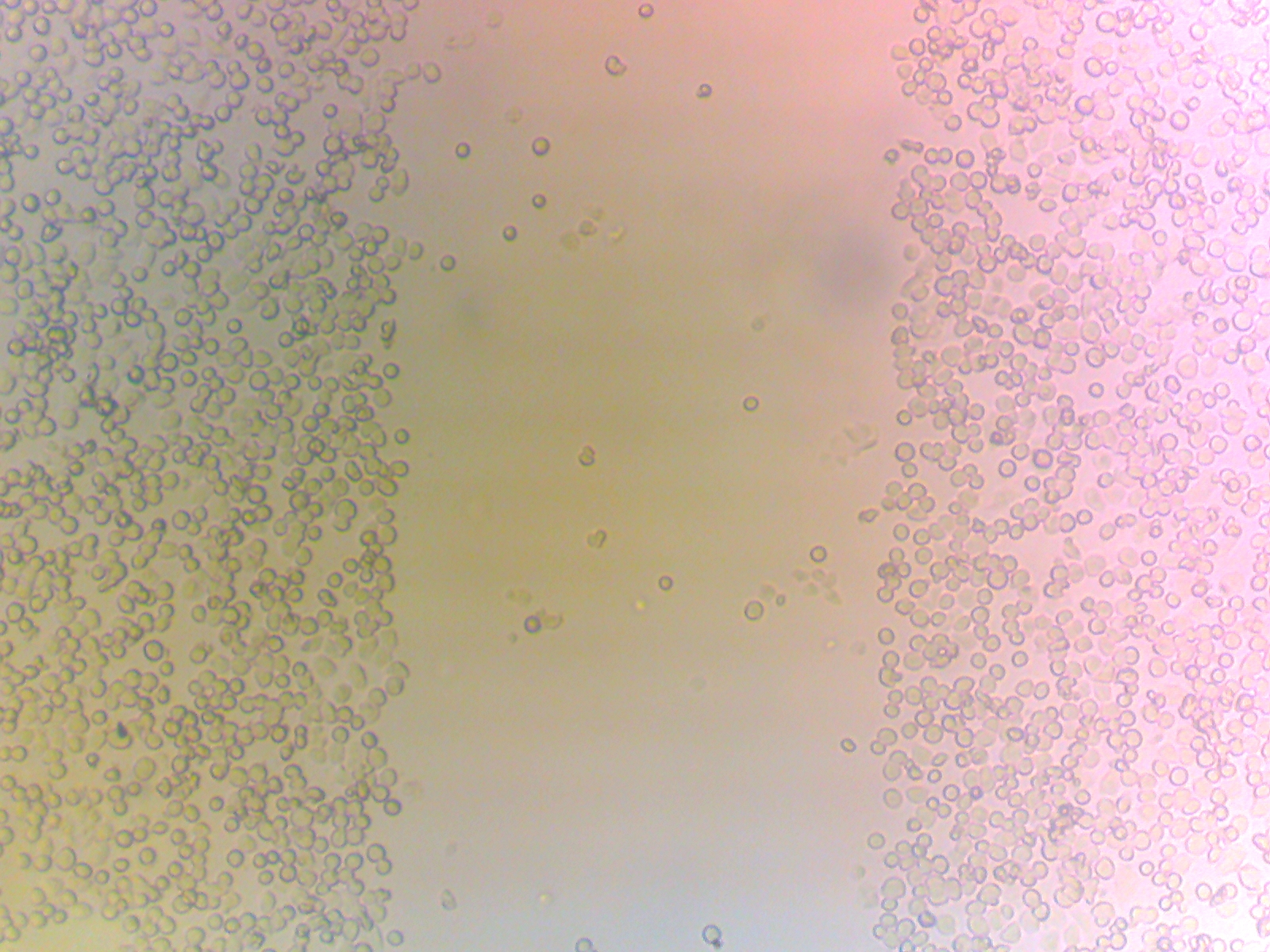

Supplement: S1 Raw data — (ZIP) [file pone.0272781.s001.zip › Raw Data_Bouchmaa et al/Migration assay/Migration assay-revision-dios and proto/Cneg/T0 Puits 'c neg rep3.jpg]
